# Scaling up HIV viral load – lessons from the large‐scale implementation of HIV early infant diagnosis and CD4 testing

**DOI:** 10.1002/jia2.25008

**Published:** 2017-11-12

**Authors:** Trevor Peter, Clement Zeh, Zachary Katz, Ali Elbireer, Bereket Alemayehu, Lara Vojnov, Alex Costa, Naoko Doi, Ilesh Jani

**Affiliations:** ^1^ Clinton Health Access Initiative Gaborone Botswana; ^2^ United States Centers for Disease Control Addis Ababa Ethiopia; ^3^ Foundation for Innovative New Diagnostics Geneva Switzerland; ^4^ African Society for Laboratory Medicine Addid Ababa Ethiopia; ^5^ ICAP University of Columbia New York USA; ^6^ World Health Organization Geneva Switzerland; ^7^ UNICEF New York USA; ^8^ Institut Nacional Da Saude Maputo Mozambique

**Keywords:** HIV, viral load, early infant diagnosis, EID, CD4 implementation

## Abstract

**Introduction:**

The scale‐up of effective HIV viral load (VL) testing is an urgent public health priority. Implementation of testing is supported by the availability of accurate, nucleic acid based laboratory and point‐of‐care (POC) VL technologies and strong WHO guidance recommending routine testing to identify treatment failure. However, test implementation faces challenges related to the developing health systems in many low‐resource countries. The purpose of this commentary is to review the challenges and solutions from the large‐scale implementation of other diagnostic tests, namely nucleic‐acid based early infant HIV diagnosis (EID) and CD4 testing, and identify key lessons to inform the scale‐up of VL.

**Discussion:**

Experience with EID and CD4 testing provides many key lessons to inform VL implementation and may enable more effective and rapid scale‐up. The primary lessons from earlier implementation efforts are to strengthen linkage to clinical care after testing, and to improve the efficiency of testing. Opportunities to improve linkage include data systems to support the follow‐up of patients through the cascade of care and test delivery, rapid sample referral networks, and POC tests. Opportunities to increase testing efficiency include improvements to procurement and supply chain practices, well connected tiered laboratory networks with rational deployment of test capacity across different levels of health services, routine resource mapping and mobilization to ensure adequate resources for testing programs, and improved operational and quality management of testing services. If applied to VL testing programs, these approaches could help improve the impact of VL on ART failure management and patient outcomes, reduce overall costs and help ensure the sustainable access to reduced pricing for test commodities, as well as improve supportive health systems such as efficient, and more rigorous quality assurance. These lessons draw from traditional laboratory practices as well as fields such as logistics, operations management and business.

**Conclusions:**

The lessons and innovations from large‐scale EID and CD4 programs described here can be adapted to inform more effective scale‐up approaches for VL. They demonstrate that an integrated approach to health system strengthening focusing on key levers for test access such as data systems, supply efficiencies and network management. They also highlight the challenges with implementation and the need for more innovative approaches and effective partnerships to achieve equitable and cost‐effective test access.

## 1 Introduction

Since 2013, when the World Health Organization strongly recommended the use of HIV viral load (VL) testing to monitor viral suppression in persons on antiretroviral therapy (ART) in low resource settings (LRS), the scale‐up of this diagnostic test in low‐ and middle‐income countries has been limited 1, 2. Of the estimated 19.5 million persons currently on ART in these countries, less then 40% have routine access to VL testing 3. This is despite the utility of this test as a means to monitor viral suppression, enable early detection of treatment failure, and guide appropriate ART management. Based on current projections, access to VL testing will reach only 60% by 2020 3.

Current VL technologies are complex nucleic acid assays that require sophisticated laboratory infrastructure, advanced technical skills, well‐established sample logistics, and systems for results return 4. Fostering the rapid and effective use of test results is also essential to achieve expected health outcomes, especially as the test is new, and unfamiliar to many clinicians and patients. Optimizing testing capacity, enabling health systems, and the appropriate use of test results to guide treatment is the focus of governments, implementing partners, donors, industry, and civil society 5, 6. Despite these efforts, scale up of routine VL testing has encountered delays and uneven success 2, 7, 8, which may impede the achievement of the global 2020 HIV 90‐90‐90 goals set by the Joint United Nations Programme on HIV/AIDS (UNAIDS) and the aspiration of ending AIDS by 2030 5.

The scale‐up of VL testing is not the first large‐scale implementation of an HIV‐related diagnostic test in low and middle income countries. In particular, the experience of CD4 assays and early infant HIV diagnosis (EID) testing over the past 15 years provide important lessons for VL implementation 9, 10, 11. This commentary reviews the use of EID and CD4 tests in RLS and identifies best practices, innovations and success factors to inform scale‐up of VL services in these same settings. The primary lessons from past experience are to ensure linkage to care and the efficiency of testing.

## 2 Improving VL programs

Over the past decade, public health programs in RLS have introduced and scaled up HIV early infant diagnosis and CD4 testing within many of the same laboratories and health facilities that are currently launching HIV VL testing. Testing coverage and volumes were initially modest – a few hundred thousand tests – but reached millions of tests within a few years 3. By 2015, an estimated 60% of those eligible for CD4 testing had access to the service, and approximately 43% of infants had access to EID testing 12, 13. Unfortunately, there is also significant evidence that many test results are not delivered to patients and/or are not used to make the necessary clinical decisions. In other words, despite significant progress, there remains a long way to go before people living with HIV (PLHIV) in RLS have universal access to high‐quality CD4 and EID testing services.

It is expected that over 20 million people will need access to VL testing by 2020 3. As VL services will be implemented at the same health facilities and laboratories as CD4 and EID, often utilizing the same staff, infrastructure, logistics, supply chains and data systems, applying lessons learned from CD4 and EID programs may accelerate scale‐up of effective VL testing services (Table 1).

**Table 1:** Key challenges and potential solutions for the implementation of HIV viral load testing in resource limited settings

Ensure linkage to care	Faster clinical decision‐making	Rapid test turn around: Faster sample transport networks Electronic result delivery Point‐of‐care testing
	Improved clinical follow‐up through the cascade of care	Health data systems: Data dashboards Dedicated patient identifiers Electronic patient medical records Electronic clinic data management and scheduling
Improve testing efficiency	Supply chain best practices	Price‐per‐result supply terms including: Instruments Reagents Service and maintenance Controls Test failures Distribution
	Tiered diagnostic network	Integrated sample referral network: Covers all clinic sites Incorporates all test types Regular frequency Low cost
	Resource mobilization	Forecasting and budgeting: Forecast national test demand Map testing funding and identify gaps Update national budgets Incorporate resource needs for lab systems strengthening
	Management	Build a management team focused on diagnostics service delivery: Operations management team Quality management team

## 3 Ensure linkage to care

CD4 and EID implementation efforts have been heavily focused on building and equipping laboratories, training staff, and procuring test commodities. These activities were often highly successful, and both CD4 and EID laboratory capacity greatly exceeded test demand in many countries 9, 10. The same is true for VL testing; in 2014, VL test volumes in sub‐Saharan Africa outside of South Africa were at 10% of the estimated 2 million test capacity of laboratories in this region 14. The set‐up of laboratories often occurs faster then health facilities can implement the clinical procedures needed to adopt and use the new tests. Because testing volumes, turn‐around‐time and costs have been the most common metrics for evaluating EID and CD4 programs, a striking fact is often overlooked – many test results are never delivered to patients and used to guide patient care. In sub‐Saharan Africa, for example an estimated 50% of CD4 and EID test results were not used 15. Common barriers to the effective utilization of test results include long test turn‐around times leading to loss to follow up, the lack of health data systems, and facility‐level operational challenges described elsewhere in this Supplement 16, 17, 18, 19, 20, 21, 22, 23.

### 3.1 Rapid sample referral and result delivery

Long test turnaround times (TAT) are particularly devastating in the case of EID, where median TAT remain long, from 20 to 60 days 24, 25. The EID cascade has been well described in a range of settings and geographies, including Africa and Asia, and highlights the challenges associated with a failure to effectively link patients to follow‐on care after testing 16, 17, 18, 19, 20. For infants tested at four to six weeks of age, EID test results are often returned after the early peak of infant HIV‐related mortality 26. TAT is also a barrier to utilization of CD4 results, and studies have shown that fewer than 50% of patients received their test results and initiated treatment promptly, when CD4 count thresholds were criteria for ART 22, 23.

Several countries have implemented improved sample referral networks and electronic result delivery to reduce TAT. This has included the mapping of sample referral routes and establishing disease‐integrated courier contracts to transport HIV, TB and other clinical specimens, e.g. with the Postal Service or Riders for Health. In Uganda, an extensive and robust sample referral network increased access to EID by 50% and reduced sample transport costs by 50% 24. Similarly, mHealth initiatives have been implemented for rapid return of test results to clinics using short message system (SMS) printers and dashboards to consolidate and present geospatial testing data to track and follow‐up on defaults to optimal TAT, for example in Kenya, Uganda, Mozambique and South Africa 24, 25, 27. The use of SMS printers for rapid result delivery has been shown to reduce TAT by nearly 50% in Uganda and Mozambique 24, 25.

### 3.2 POC diagnostics

The use of point‐of‐care (POC) diagnostic technologies significantly improves retention in the pre‐ART care cascade. POC testing significantly reduced EID TAT and increased access to ART amongst HIV‐infected infants in Mozambique 28. Similarly, POC CD4 testing reduced TAT from ten days to less than one day on average 22. In Mozambique, POC CD4 reduced loss‐to‐follow‐up before ART initiation by 50% 23. Access to POC VL testing may also help improve the management of treatment failure for patients on ART by dramatically reducing TAT and enabling faster clinical decisions, however studies on this are needed.

### 3.3 Data systems to improve the cascade of care

Other critical enablers of effective test utilization include integrated data and clinical systems that improve the cascade of care from the identification of eligible patients, the provision of testing, and delivery of results back to the health facility and patient. This enables results to be used swiftly to make clinical decisions. It is therefore important to build diagnostic systems with patient care in mind, ensuring that delivery of results to health facilities is well integrated with clinical patient management and well synchronized with the patient pathway of care. Several interventions have been shown to improve the cascade of clinical care from testing through linkage to care, including clinical quality improvements such as the use of SMS result delivery to clinics or patients, and active patient follow‐up by community health workers 29, 30, 31, 32, 33, 34, 35. In Mozambique, SMS result delivery to clinics was associated with a 20% increase in receipt of test results by caregivers, and a three‐fold decrease in testing errors 25, 36. Data systems that enable the follow‐up of patients through the cascade of care have been shown to improve overall clinical outcomes, for example in South Africa where visual dashboards enabled data‐driven interventions to improve health program performance against key indicators for the prevention of mother to child infection 27.

### 3.4 Application to VL programs

It will be useful to draw from the above interventions that have proven to be successful with CD4 and EID programs so that steps can be taken early in VL implementation to establish effective practices. In particular, testing programs need to ensure that VL testing is well integrated with ART failure management processes, for example that test TAT do not introduce long delays in detecting and confirming failure, and that results are delivered to clinicians and patients in order for timely follow‐on care, such as enhanced adherence support and/or a drug regimen switch. VL monitoring and evaluation programs should also include metrics such as testing coverage, test TAT, test delivery to clinics and patients, and test utilization to guide ART management. In many settings, VL programs can leverage existing EID and CD4 sample referral and electronic results delivery networks. The utility of POC VL still needs further investigation, but it is possible that the availability of rapid VL results may improve patient adherence, differentiated care, and reduce program costs (Table 1).

## 4 Improve testing efficiency

The establishment of large scale CD4 and EID testing programs in low‐resource settings over the past 15 years faced many challenges 9, 11. As a result the focus of these programs was often on ensuring test access. As access has improved, attention is turning to testing efficiency in order to ensure the clinical impact of testing and to reduce costs. Key interventions to improve efficiency are described below (Table 1).

### 4.1 Procurement and supply chain

Large‐scale VL testing programs will consume significant quantities of diagnostic commodities run on networks of instruments. Ensuring the reliable and efficient supply of testing products and preventing breakdowns of instruments requires some of the innovative approaches that have been developed within EID and CD4 testing programs. At the outset of these programs, test kit and instrument costs were high, test procurement was highly disaggregated across government, donors and implementing partners, and stock outs and instrument breakdowns were frequent. Many of these challenges have not yet been resolved; a recent WHO survey found approximately 10% of instruments were not in use due to stock‐outs, breakdowns or non‐installation 37. Two supply chain innovations – global access price deals and bundled agreements – have been implemented for the CD4 and EID markets, and are highly relevant to VL.

Global access price deals are negotiated centrally and provide equitable access to low pricing for all public sector buyers irrespective of procurement volume. This allows smaller countries with limited purchasing power to access the same pricing as high volume countries. Price reductions of 30% to 50% for CD4 and EID tests have resulted in substantial commodity budget savings for countries and donors around the world, and have helped expand access to these tests 38, 39. Access pricing is also available for certain VL test suppliers and this has helped make VL more accessible 40.

Supply chain initiatives can have significant impact, reducing costs and increasing health product availability 41. For diagnostics, supply terms such as all‐in or price‐per‐result (incorporating instruments, reagents, training, quality assurance, distribution and test failures), reagent rental agreements that remove up‐front instrument capital cost, and bundled reagent‐maintenance agreements that eliminate separate maintenance contracts, have been designed and implemented for EID and CD4 testing networks. These are designed to reduce costs and financial barriers, improve procurement flexibility, efficiency and supplier accountability, and reduce instrument down‐time and stock‐outs. They also establish partnerships between suppliers and procurers and encourage more coordinated procurement. Coordinated procurement, whereby testing volumes and ordering schedules across procurers and over time are consolidated to negotiate improved supplier terms, has been effectively used to improve CD4 and EID supply chains.

The Global Fund has established framework agreements with major VL suppliers that provide mechanisms for standardized access to bundled supply terms 42. In addition, several national testing programs, for example Brazil, South Africa, Kenya and Uganda, have implemented bundled agreements for VL testing. These build on procurement principles and mechanisms developed with large‐scale CD4 and EID programs. Instead of procuring instruments, testing kits, and annual instrument service and maintenance separately, national testing volumes have been consolidated (by the government or through a procurement consortium of government, donors and implementing partners), and a single contract for supply of instruments, reagents and service and other test components at prices lower than disaggregated procurement. Bundled procurement helps ensure service and maintenance is always accessible; a critical need in many countries. The recent WHO survey found that most CD4 instruments in public laboratories were not covered by service and maintenance contracts, were not serviced regularly and breakdowns were frequent 37. Bundled procurement requires well‐established forecasts and regular ordering cycles, which help ensure reliable test manufacture and supply. Bundled agreements also provide the flexibility to switch, if needed, to more competitive suppliers at the end of each contract because the instruments were not purchased. For example, bundled agreements have been used in South Africa and Brazil for over ten years and have enabled easier switches in testing technologies without having to formally retire instruments and the financial hurdle of paying for new equipment up front 14.

Although they are valuable tools for optimizing supply chain, bundled agreements have been infrequently used for VL testing programs, partly due to unconsolidated procurement or difficulty with undertaking multi‐year procurement commitments. As VL testing programs scale‐up to meet growing demand, the need for more effective procurement mechanisms and better coordinated procurement across buyers will also grow. Given the relatively high reagent and capital costs, and the technical complexity of VL instruments, and hence need for routine service by an engineer, carefully negotiated bundled agreements and access pricing will likely be useful measures to increase the sustainability and reliability of large‐scale testing programs.

### 4.2 Laboratory networks

Health systems in many LRS often have limited ability to support large testing networks 43, 44. In particular, instrument technology‐based testing programs require substantial supportive systems. To ensure widespread access, test networks are required, comprising a tiered network of laboratories servicing a wider network of health facilities. The functioning of such networks relies heavily on testing systems such as sample referral to ensure access to laboratory capacity over wide geographic areas, health data systems to ensure rapid dissemination of test results for clinical, programmatic and surveillance uses, and quality assurance to ensure reliable results irrespective of testing location 45, 46. Laboratory networks have been strengthened in numerous countries, establishing active sample referral and results delivery connections between central and decentralized levels of the health care system. For example, Mali, Burkina Faso and Senegal through the Resaolab program have improved surveillance and outbreak detection readiness, in line with the eight WHO laboratory requirements of the International Health Regulations and laboratory readiness requirements of the Global Health Security Agenda 47.

The rational deployment of testing technologies within the networks is a key factor for efficiency. A common feature of CD4 and EID testing networks to date has been over‐capacity and under‐utilization of the instrument base. A 2013 WHO survey in 127 countries identified a surplus of global CD4 capacity, sufficient instrumentation to conduct 4.6 tests per year for every person living with HIV, and 12.8 tests/year for every person on ART, as opposed to the 1 to 2 tests per year required at that time 37. Capacity utilization across instruments was only 13.7%. At the start of scale‐up programs it was likely common to deploy excess instruments to ensure no capacity limitations. As testing programs mature, there are opportunities to build more efficient networks and to increase instrument utilization. The lessons for VL deployment are to start testing programs by placing devices with appropriate capacity at each testing location. For example, placing high throughput instruments at central test demand locations and lower throughput devices at smaller sites with robust sample referral linkages that maximize instrument utilization and therefore, the return on the investment made for each instrument 48. This requires data on testing current and projected needs across the network of health facilities, the mapping of sample referral routes, and coordinated national instrument procurement and deployment plans 24, 49. Several tools now exist to help optimize testing networks, including the USAID‐developed LabEQIP tool 50.

### 4.3 Resource mobilization

One of the key lessons from the scale‐up of CD4 and EID programs is while significant donor funding has gone into laboratory services over the past decade, long‐term national investment in laboratory testing is needed. Effective national laboratory systems such as logistic and health data systems are often under‐funded and not fully part of routine service. This is partly because the full costs of testing services, especially when new diagnostics are being rapidly scaled up, and the financial resources needed to ensure access to essential diagnostic tests at national scale are difficult to estimate and track. As a result, large‐scale public testing programs such as VL face risks and may be unstable and unsustainable unless appropriate investments are made in testing and necessary supportive systems. For example, in Zimbabwe despite a goal of 21% VL coverage in 2015, only 5.6% test coverage was achieved due to challenges in resource mobilization and related factors 49. For fast growing testing programs such as VL, test projections need to routinely inform national laboratory budgets to reduce the risk of funding shortfalls. Data systems such as test dashboards, that are connected to laboratory and POC instruments, can track test volumes and utilization in real time and produce consumption data to better inform national HIV diagnostics quantification exercises, for example using the USAID‐developed ForLab tool 50.

In addition, the investment case for laboratory systems strengthening needs to be more clearly articulated so that sustainable funding for these systems can be established. Investments in scaling up CD4 and EID in many countries have often included laboratory system strengthening programs for logistics, data and quality, providing an opportunity to build on these investments to support VL testing programs 9. Guidelines of the Global Fund clearly elaborate a comprehensive range of critical laboratory strengthening investments that countries can make using Global Fund resources, including human resource capacity development, procurement and equipment management systems, quality improvement, development of tiered laboratory networks, and data systems 51.

### 4.4 Management

The unprecedented scale of CD4 and EID testing programs has highlighted the need for stronger management within public laboratory programs, both operational and quality management. Laboratory quality improvement programs such as the Strengthening Laboratory Management Towards Accreditation (SLMTA), developed by the United States Centers for Disease Control and Prevention, and the WHO Strengthening Laboratory Improvement Towards Accreditation (SLIPTA) program supported by the African Society for Laboratory Medicine have increased quality management skills and have established a growing trend towards accreditation in public laboratories 52. Countries such as India, Ethiopia, Ghana, Nigeria, and the Caribbean region have made significant steps towards strengthening quality management skills and laboratory accreditation 53, 54, 55 Although quality improvement systems are still to fully establish across a wider range of countries, these quality initiatives provide a foundation that VL programs will be able to leverage to ensure reliable testing.

Tiered networks of laboratories have significant operational management needs to ensure efficiency; however most leaders of public health laboratory networks are not trained in operations 56. This includes the infrastructure management, i.e. ensuring adequate physical space and equipment within laboratories, financial management including budgeting, supply chain and distribution systems, logistics and data systems, and monitoring and evaluation. Improved operations management within laboratory networks may improve adherence to performance standards and ensure a service‐oriented relationship with health facilities and other stakeholders in testing services. Many of the systems weaknesses observed with CD4 and EID scale‐up and described above, such as gaps in supply chain, slow sample referral and test turn around times, and limited use of data systems may be related to inadequate operations management capacity and skills. The strengthening of this capacity within public departments responsible for laboratory services may help ensure more effective delivery of diagnostic tests within the parameters needed for clinical management and improved patient outcomes.

## 5 Conclusions

The global scale‐up of EID and CD4 testing services has established important systems and experience with large‐scale public health testing programs. It has also identified weaknesses in diagnostic access that may ultimately limit the success of these programs, as well as new tests such as VL. There are many innovations and best practices from these initial large‐scale testing programs to learn from, as well as persistent challenges to overcome. There are critical needs and opportunities in linkage to care and testing efficiency, including improved data and sample referral systems, POC tests, improved supply terms, and more effective laboratory network management that are important to address during the scale‐up of HIV VL testing programs in resource limited settings (Table 1). Learning from prior experiences with CD4 and EID test scale‐up will help ensure accessible and reliable VL services and help overcome major test implementation barriers.

## 6 Competing interests

The authors have no conflicts of interest.

## 7 Authors’ contributions

TP, CZ, ZK, AE, BA, LV, AC, ND and IJ developed and finalized the manuscript.

## 9 Funding

Unitaid, PEPFAR.

## References

[1] World Health Organization . Consolidated Guidelines on the Use of Antiretroviral Drugs for Treating and Preventing HIV Infection. Recommendations for a Public Health Approach. 2nd ed. Geneva, Switzerland: World Health Organization; 2016. 27466667

[2] Lecher S , Williams J , Fonjungo PN , Kim AA , Ellenberger D , Zhang G . Progress with Scale‐Up of HIV Viral Load Monitoring – Seven Sub‐Saharan African Countries, January 2015‐June. MMWR Morb Mortal Wkly Rep. 2016;65:1332–5. 27906910 10.15585/mmwr.mm6547a2

[3] World Health Organization . HIV diagnostic tests in low‐ and middle‐income countries: forecasts of global demand for 2014–2018. Geneva, Switzerland: World Health Organization; 2015.

[4] UNITAID . HIV/AIDS diagnostics technology landscape. 5th ed Geneva, Switzerland: UNITAID; 2015.

[5] Joint United Nations Programme on HIV/AIDS . 90‐90‐90 an ambitious treatment target to help end the AIDS epidemic. 2014 Oct; Available from: http://www.unaids.org/sites/default/files/media_asset/90-90-90_en_0.pdf.

[6] Peter T , Ellenberger D , Kim AA , Boeras D , Messele T , Roberts T , et al. Early antiretroviral therapy initiation: access and equity of viral load testing for HIV treatment monitoring. Lancet Infect Dis. 2017;17(1):e26–9. 27773596 10.1016/S1473-3099(16)30212-2 PMC5745573

[7] Roberts T , Cohn J , Bonner K , Hargreaves S . Scale‐up of routine viral load testing in resource‐poor settings: current and future implementation challenges. Clin Infect Dis. 2016;62:1043–8. 26743094 10.1093/cid/ciw001 PMC4803106

[8] Carmona S , Peter T , Berrie L . HIV viral load scale‐up: multiple interventions to meet the HIV treatment cascade. Curr Opin HIV AIDS. 2017;12:157–64. 28134713 10.1097/COH.0000000000000352

[9] Peter T , Badrichani A , Wu E , Freeman R , Ncube B , Ariki F , et al. Challenges in implementing CD4 testing in resource‐limited settings. Cytometry B Clin Cytom. 2008;74 Suppl 1:S123–30. 18348208 10.1002/cyto.b.20416

[10] Diallo K , Kim AA , Lecher S , Ellenberger D , Beard RS , Dale H . Early Diagnosis of HIV Infection in Infants ‐ One Caribbean and Six Sub‐Saharan African Countries, 2011‐2015. MMWR Morb Mortal Wkly Rep. 2016;65(46):1285–90. 27880749 10.15585/mmwr.mm6546a2

[11] Essajee S. , Bhairavabhotla R , Penazzato M , Kiragu K , Jani I , Carmona S , et al. Scale‐up of Early Infant HIV Diagnosis and Improving Access to Pediatric HIV Care in Global Plan Countries: Past and Future Perspectives. J Acquir Immune Defic Syndr. 2017;75:S51–8. 28398997 10.1097/QAI.0000000000001319

[12] Joint United Nations Programme on HIV/AIDS . Global plan towards the elimination of new HIV infections among children by 2015 and keeping their mothers alive 2011–2015. Geneva: UNAIDS; 2016.

[13] Joint United Nations Programme on HIV/AIDS . On fast track to an AIDS‐free generation. 2016 Available from: http://www.unaids.org/sites/default/files/media_asset/GlobalPlan2016_en.pdf.

[14] Stevens W . Viral load testing in Africa: 23 years later. Lessons learned, future challenges and opportunities. Presentation at the African Society for Laboratory meeting on HIV Viral Load Implementation in Africa, March 2013.

[15] UNICEF . Children and AIDS: a stocktaking report. New York: UNICEF; 2007 http://www.unicef.org/publications/files/ChildrenanAIDSAStocktakingLoResPDF_EN_USLetter_15012007.pdf.

[16] Chatterjee A , Tripathi S , Gass R , Hamunime N , Panha S , Kiyaga C , et al. Implementing services for Early Infant Diagnosis (EID) of HIV: a comparative descriptive analysis of national programs in four countries. BMC Public Health. 2011;11:553–62. 21749730 10.1186/1471-2458-11-553 PMC3161890

[17] Hsiao N‐Y , Stinson K , Myer L . Linkage of HIV‐infected infants from diagnosis to antiretroviral therapy services across the Western Cape, South Africa. PLoS ONE. 2013;8:e55308. 23405133 10.1371/journal.pone.0055308 PMC3566187

[18] Rawizza HE , Chang CA , Chaplin B , Ahmed IA , Meloni ST , Oyebode T , et al. Loss to follow‐up within the prevention of mother‐to‐child transmission care cascade in a large art program in Nigeria. Curr HIV Res. 2015;13:201–9. 25986371 10.2174/1570162x1303150506183256 PMC5495655

[19] Sibanda EL , Weller IV , Hakim JG , Cowan FM . The magnitude of loss to follow‐up of HIV‐exposed infants along the prevention of mother‐to‐child HIV transmission continuum of care: a systematic review and meta‐analysis. AIDS. 2013;27:2787–97. 24056068 10.1097/QAD.0000000000000027 PMC3814628

[20] Sirirungsi W , Khamduang W , Collins IJ , Pusamang A , Leechanachai P , Chaivooth S , et al. Early infant HIV diagnosis and entry to HIV care cascade in Thailand: an observational study. Lancet HIV. 2016;3:e259–65. 10.1016/S2352-3018(16)00045-X. 27240788 PMC6047735

[21] Vermund SH , Blevins M , Moon TD , José E , Moiane L , Tique JA , et al. Poor clinical outcomes for HIV infected children on antiretroviral therapy in rural Mozambique: need for program quality improvement and community engagement. PLoS ONE. 2014;9:e110116. 25330113 10.1371/journal.pone.0110116 PMC4203761

[22] Vojnov L , Markby J , Boeke C , Harris L , Ford N , Peter T . Point‐of‐care CD4 Testing improves linkage to HIV care and timeliness of ART initiation in a public health approach: a systematic review and meta‐analysis. PLoS ONE. 2016;11:e0155256. 27175484 10.1371/journal.pone.0155256 PMC4866695

[23] Jani IV , Sitoe NE , Alfai ER , et al. Effect of point‐of‐care CD4 cell count tests on retention of patients and rates of antiretroviral therapy initiation in primary health clinics: an observational cohort study. Lancet. 2011;378:1572–9. 21951656 10.1016/S0140-6736(11)61052-0

[24] Kiyaga C , Sendagire H , Joseph E , McConnell I , Grosz J , Narayan V , et al. Uganda's new national laboratory sample transport system: a successful model for improving access to diagnostic services for Early Infant HIV Diagnosis and other programs. PLoS ONE. 2013;8(11):e78609. 24236026 10.1371/journal.pone.0078609 PMC3827263

[25] Deo S , Crea L , Quevedo J , Lehe J , Vojnov L , Peter T , et al. Implementation and operational research: expedited results delivery systems using GPRS technology significantly reduce early infant diagnosis test turnaround times. J Acquir Immune Defic Syndr. 2015;70(1):e1–4. 26068719 10.1097/QAI.0000000000000719

[26] Bourne DE , Thompson M , Brody LL , Cotton M , Draper B , Laubscher R , et al. Emergence of a peak in early infant mortality due to HIV/AIDS in South Africa. AIDS. 2009;23:101–6. 19065753 10.1097/qad.0b013e32831c54bd

[27] Bhardwaj S , Barron P , Pillay Y , Treger‐Slavin L , Robinson P , Goga A , et al. Elimination of mother‐to‐child transmission of HIV in South Africa: rapid scale‐up using quality improvement. S Afr Med J. 2014;104 3 Suppl 1:239–43. 24893500 10.7196/samj.7605

[28] Jani IV , Meggi B , Loqiha O , Tobaiwa O , Mudenyanga C , Zitha A , et al. Effect of point‐of‐care early infant diagnosis on retention of patients and rates of antiretroviral therapy initiation in primary health care clinics: a cluster‐randomised trial in Mozambique. Abstract presented at CROI, Seattle, February 2017.

[29] Creek TL , Sherman GG , Nkengasong J , Lu L , Finkbeiner T , Fowler MG , et al. Infant human immunodeficiency virus diagnosis in resource‐limited settings: issues, technologies, and country experiences. Am J Obstet Gynecol. 2007;197:S64–71. 17825652 10.1016/j.ajog.2007.03.002

[30] Penazzato M , Revill P , Prendergast AJ , Collins IJ , Walker S , Elyanu PJ , et al. Early infant diagnosis of HIV infection in low‐income and middle‐income countries: does one size fit all? Lancet Infect Dis. 2014;14:650–5. 24456814 10.1016/S1473-3099(13)70262-7

[31] Cohn J , Whitehouse K , Tuttle J , Lueck K , Tran T . Paediatric HIV testing beyond the context of prevention of mother‐to‐child transmission: a systematic review and meta‐analysis. Lancet HIV. 2016;3:e473–81. 27658876 10.1016/S2352-3018(16)30050-9

[32] Essajee S , Vojnov L , Penazzato M , Jani I , Siberry GK , Fiscus SA , et al. Reducing mortality in HIV‐infected infants and achieving the 90‐90‐90 target through innovative diagnosis approaches. J Int AIDS Soc. 2015;18:20299. 26639120 10.7448/IAS.18.7.20299 PMC4670838

[33] Ciaranello A , Park J , Ramirez‐Avila L , Freedberg KA , Walensky RP , Leroy V . Early infant HIV‐1 diagnosis programs in resource‐limited settings: opportunities for improved outcomes and more cost‐effective interventions. BMC Med. 2011;9:59–64. 21599888 10.1186/1741-7015-9-59 PMC3129310

[34] Diallo K , Modi S , Hurlston M , Beard RS , Nkengasong JN . A Proposed Framework for the Implementation of Early Infant Diagnosis Point‐of‐Care. AIDS Res Hum Retroviruses. 2017;33(3):203–10. 27758117 10.1089/aid.2016.0021 PMC5333568

[35] Chibwesha CJ , Chi BH . Expanding coverage of paediatric HIV testing. Lancet HIV. 2016;3:e451–3. 27658877 10.1016/S2352-3018(16)30064-9

[36] Jani IV , Quevedo JI , Tobaiwa O , Bollinger T , Sitoe NE , Chongo P , et al. Use of mobile phone technology to improve the quality of point‐of‐care testing in a low‐resource setting. AIDS. 2016;30:159–61. 26731762 10.1097/QAD.0000000000000907

[37] Habiyambere V , Ford N , Low‐Beer D , Nkengasong J , Sands A , Pérez González M . Availability and use of HIV monitoring and early infant diagnosis technologies in WHO member states in 2011–2013: analysis of annual surveys at the facility level. PLoS Med. 2016;13(8):e1002088. 27551917 10.1371/journal.pmed.1002088 PMC4995037

[38] Joint United Nations Programme on HIV/AIDS . Breakthrough global agreement sharply lowers price of early infant diagnosis of HIV. Geneva: UNAIDS; 2015 Available from: http://www.unaids.org/en/resources/presscentre/pressreleaseandstatementarchive/2015/july/20150719_EID_pressrelease.

[39] Clinton Foundation . Landmark Agreement on HIV/AIDS Lab Test Price Reduction. 2004 Available from: https://www.clintonfoundation.org/main/news-and-media/press-releases-and-statements/press-release-landmark-agreement-on-hiv-aids-lab-test-price-reduction.html.

[40] Joint United Nations Programme on HIV/AIDS . Landmark HIV Diagnostic Access Program Will Save $150M And Help Achieve Global Goals On HIV. 2014 Available from: http://www.unaids.org/en/resources/presscentre/pressreleaseandstatementarchive/2014/september/20140925prviralload.

[41] Seidman G , Atun R . Do changes to supply chains and procurement processes yield cost savings and improve availability of pharmaceuticals, vaccines or health products? A systematic review of evidence from low‐income and middle‐income countries. BMJ Glob Health. 2017;2:e000243. 10.1136/bmjgh-2016-000243 PMC5435270 28589028

[42] The Global Fund . HIV viral load and early infant diagnosis product selection and procurement tool. Geneva, Switzerland; 2017 Available from: https://www.theglobalfund.org/media/5765/psm_viralloadearlyinfantdiagnosis_content_en.pdf.

[43] Nkengasong JN . Strengthening laboratory services and systems in resource‐poor countries. Am J Clin Pathol. 2009;131(6):774. 19461081 10.1309/AJCP8GYX8KTKDATZ

[44] Alemnji GA , Zeh C , Yao K , Fonjungo PN . Strengthening national health laboratories in sub‐Saharan Africa: a decade of remarkable progress. Trop Med Int Health. 2014;19(4):450–8. 24506521 10.1111/tmi.12269 PMC4826025

[45] Fonjungo PN , Alemnji GA , Kebede Y , Opio A , Mwangi C , Spira TJ . Combatting global infectious diseases: a network effect of specimen referral systems. Clin Infect Dis. 2017;64:796–803. 28200031 10.1093/cid/ciw817

[46] Kiyaga C , Sendagire H , Joseph E , Grosz J , McConnell I , Narayan V , Esiru G , et al. Consolidating HIV testing in a public health laboratory for efficient and sustainable early infant diagnosis (early infant diagnosis) in Uganda. J Public Health Policy. 2015;36:153–69. 25811386 10.1057/jphp.2015.7

[47] Najjar‐Pellet J , Machuron JL , Bougoudogo F , Sakandé J , Sow I , Paquet C , et al. Clinical laboratory networks contribute to strengthening disease surveillance: the RESAOLAB Project in West Africa. Emerg Health Threats J. 2017;6:1,19960. 10.3402/ehtj.v6i0.19960 PMC3557952 23362415

[48] Williams J , Umaru F , Edgil D , Kuritsky J . Progress in harmonizing tiered HIV laboratory systems: challenges and opportunities in 8 African countries. Glob Health Sci Pract. 2016;29(4):467–80. 10.9745/GHSP-D-16-00004 PMC5042701 27688718

[49] Kilmarx PH , Simbi R . Progress and challenges in scaling up laboratory monitoring of HIV treatment. PLoS Med. 2016;13(8):e1002089. 27551962 10.1371/journal.pmed.1002089 PMC4995050

[50] USAID . ForLab Laboratory Quantification Tool and LabEQIP Software Tool. Available from: https://www.ghsupplychain.org/resources/other-resources.

[51] The Global Fund . Technical Brief: Strategic Support for Integrated Laboratory Services. Geneva, Switzerland; 2016 Available from: https://www.theglobalfund.org/media/5532/core_laboratoryservices_technicalbrief_en.pdf.

[52] ASLM . SLIPTA Audited Laboratories Distribution Map. 2017 Available from: http://www.aslm.org/resource-centre/slipta-map/.

[53] Yao K , Maruta T , Luman ET , Nkengasong JN . The SLMTA programme: Transforming the laboratory landscape in developing countries. Afr J Lab Med. 2014 Available at: http://www.ajlmonline.org/index.php/ajlm/article/view/194/182. 10.4102/ajlm.v3i2.194 PMC4703335 26752335

[54] Guevara G , Gordon F , Irving Y , Whyms I , Parris K , Beckles S , et al. The impact of SLMTA in improving laboratory quality systems in the Caribbean Region. Afr J Lab Med. 2014;3(2):199. 27066396 10.4102/ajlm.v3i2.199 PMC4826060

[55] Nkrumah B , van der Puije B , Bekoe V , Adukpo R , Kotey NA , Yao K , et al. Building local human resources to implement SLMTA with limited donor funding: the Ghana experience. Afr J Lab Med. 2014 Available at: http://www.ajlmonline.org/index.php/ajlm/article/view/214/207. 10.4102/ajlm.v3i2.214 PMC4770820 26937417

[56] Alemnji G , Onyebujoh P , Nkengasong JN . Improving laboratory efficiencies to scale‐up HIV viral load testing. Curr Opin HIV AIDS. 2017;12(2):165–70. 28059956 10.1097/COH.0000000000000346 PMC13081754

